# Comparison of Two Sources of Clinical Audit Data to Assess the Delivery of Diabetes Care in Aboriginal Communities

**DOI:** 10.3390/ijerph14101236

**Published:** 2017-10-17

**Authors:** Timothy Regan, Christine Paul, Paul Ishiguchi, Catherine D’Este, Claudia Koller, Kristy Forshaw, Natasha Noble, Christopher Oldmeadow, Alessandra Bisquera, Sandra Eades

**Affiliations:** 1 School of Medicine and Public Health, The University of Newcastle, Callaghan, NSW 2308, Australia; timothy.regan@newcastle.edu.au (T.R.); claudia.koller1@gmail.com (C.K.); kristy.forshaw@newcastle.edu.au (K.F.); natasha.noble@newcastle.edu.au (N.N.); abisquera@gmail.com (A.B.); 2 The Hunter Medical Research Institute, New Lambton Heights, NSW 2305, Australia; Christopher.Oldmeadow@hmri.org.au; 3 Baker IDI Heart and Diabetes Institute, Melbourne, VIC 3004, Australia; pishiguchi@yahoo.com (P.I.); Sandra.Eades@baker.edu.au (S.E.); 4 Department of Epidemiology and Preventive Medicine, Monash University, Melbourne, VIC 3004, Australia; 5 National Centre for Epidemiology and Population Health, Research School of Population Health, Australian National University, Canberra, ACT 0200, Australia; catherine.deste@anu.edu.au

**Keywords:** decision support systems, clinical, community health services, information storage and retrieval, testing and monitoring

## Abstract

The objective of this study was to determine the concordance between data extracted from two Clinical Decision Support Systems regarding diabetes testing and monitoring at Aboriginal Community Controlled Health Services in Australia. De-identified PenCAT and Communicare Systems data were extracted from the services allocated to the intervention arm of a diabetes care trial, and intra-class correlations for each extracted item were derived at a service level. Strong to very strong correlations between the two data sources were found regarding the total number of patients with diabetes per service (Intra-class correlation [ICC] = 0.99), as well as the number (ICC = 0.98–0.99) and proportion (ICC = 0.96) of patients with diabetes by gender. The correlation was moderate for the number and proportion of Type 2 diabetes patients per service in the group aged 18–34 years (ICC = 0.65 and 0.8–0.82 respectively). Strong to very strong correlations were found for numbers and proportions of patients being tested for diabetes, and for appropriate monitoring of patients known to have diabetes (ICC = 0.998–1.00). This indicated a generally high degree of concordance between whole-service data extracted by the two Clinical Decision Support Systems. Therefore, the less expensive or less complex option (depending on the individual circumstances of the service) may be appropriate for monitoring diabetes testing and care. However, the extraction of data about subgroups of patients may not be interchangeable.

## 1. Introduction

The complexity of managing patient healthcare needs has led to increasing utilisation of eHealth initiatives, including Clinical Decision Support Systems (CDSSs) [1,2,3]. CDSSs allow users to generate reports regarding the delivery of services in their clinic, such as the frequency of blood testing, which can be used to identify areas needing improvement in healthcare delivery. Various systematic reviews [1] indicate that CDSSs are effective in changing clinician behaviour [2], enhancing clinician adherence to clinical practice guidelines [3,4,5], and facilitating goal-setting for improving healthcare. The effectiveness of CDSSs in improving processes of care for dyslipidaemia and diabetes in primary care is well established [6,7], although issues of safety, cost, and provider satisfaction have not been rigorously investigated [6]. Unintended consequences of CDSSs include difficulty in keeping content current, inappropriate content, alert fatigue, and potential for errors [8]. CDSSs may even be effective at changing provider behaviour despite provider ambivalence [9].

The prevalence of diabetes among Indigenous Australians is estimated to be between 3 to 5 times higher than non-Indigenous Australians, ranging from 5% among those aged 25–34 to 39% among those aged 55 years and older. Indigenous Australians are more likely to live with diabetes-related complications and 10 to 13 times more likely to die from diabetes [10]. Given its chronic nature and rising prevalence, the management and prevention of Type 2 diabetes requires a consistent, multidisciplinary approach to care [11]. This involves the integration of data regarding the testing of at-risk individuals and the monitoring of patients already diagnosed. However, rates of testing and monitoring among Indigenous populations are not optimal [11]. Thus, clinicians and clinical services can benefit from the use of CDSSs to assess gaps in their service to ensure delivery of optimal diabetes care.

The utility of CDSSs depends on varying features, including cost, data flexibility, and the automation of reports and decision support tools. In this study, we explored two particular CDSSs used in Aboriginal Community Controlled Health Services (ACCHSs) in Australia: Communicare and PenCAT. Communicare is an integrated electronic health and practice management system that allows the collection and maintenance of detailed clinical information, along with tools for tasks such as the management of waiting lists and appointment coordination. Communicare can be used as the central patient management software tool and can track patients over time and generate a range of user-defined patient- and service-level reports. PenCAT is a software tool that is compatible with a number of practice management programs, and can aggregate patient information at a practice level, displaying information in a series of reports, graphs, and figures that can be used to assess whole-of-practice care performance. The primary difference between the use of these two tools is that the PenCAT tool lacks the capacity to track unique patients over time, but does generate service-level reports and is typically a lower cost option than Communicare.

### Objectives

The objectives of this study were to determine the level of concordance between data extracted from PenCAT and Communicare systems regarding the following at each ACCHS:

1. Patients with a diagnosis of Type 2 diabetes at each service, by total size, gender, and age-group.
2. Number of patients aged 35 years or over without a diagnosis of Type 2 diabetes who were tested for Type 2 diabetes via a random plasma glucose test HbA1c in a 12-month period.
3. Patients aged 18 years or over with a diagnosis of Type 2 diabetes who had their HbA1c monitored in a six-month period.
4. Patients aged 18 years or over with a diagnosis of Type 2 diabetes who had their cholesterol monitored in a 12-month period.

The items selected for comparison related to performance against diabetes-related guidelines which were current at the time of data collection [12,13,14,15,16]. The guidelines differ in recommending testing for diabetes among Aboriginal and Torres Strait Islander people every one or three years for all those aged 18 years and over; or every three years for all those aged 35 years and over.

## 2. Materials and Methods

### 2.1. Design: Cross-Sectional Study

This study used baseline data collected as part of the Achieving Diabetes Action and Collaborative Change study (ADACC). ADACC is a cluster randomised controlled trial of a systems-based intervention to improve rates of diabetes testing, monitoring, and control within 18 Aboriginal Community Controlled Health Services (ACCHSs; nine intervention sites; nine control sites). This study includes data only extracted from intervention sites during the trial baseline period.

### 2.2. Participants

#### 2.2.1. ACCHSs

Eligible sites for ADACC were those that used Communicare practice management software, maintained an electronic data management system for pathology results, and employed at least one general practitioner. Eighteen ACCHSs agreed to participate in the ADACC study and were diverse, covering metropolitan, rural, and remote areas; as well as varying total patient volume. The current study obtained data from the nine intervention sites that were assisted to implement PenCAT reporting software, in order to facilitate the generation of specific ACCHS diabetes care audits throughout the ADACC trial.

#### 2.2.2. Patients

Eligible patients were those that were defined as being ‘current’ patients in the Communicare and PenCAT systems and had visited an ACCHS at least three times in the previous two years, including at least one visit in the previous 12 months. Patients with diabetes were defined as those who have been diagnosed with Type 2 diabetes prior to or during the ADACC study baseline period. Non-diabetic patients were those with no known diabetes prior to the baseline period. Individual patient consent was waived.

#### 2.2.3. Data Collection

PenCAT data were extracted by administrative staff at each site. These data were received as a service-level summary for each item. Communicare data for all sites were extracted remotely by a Communicare technician and forwarded to the research team. Communicare data were received as de-identified individual unit record data.

#### 2.2.4. Data Analysis

Intra-class correlations (ICCs) for the measures listed above were derived from a simple two-level hierarchical linear model, where the first level consists of the service-level means modelled as a normally distributed random effect (with a constant between-group variance), and the second level consists of the measurements from the two different systems (PenCAT or Communicare) assumed to be normally distributed conditional on the service mean (with a constant within-group variance). The ICCs were estimated as the ratio of the between-service variance over the total variance (the sum of the within- and between-service variances). Due to the low number of sites, ICCs were estimated using Bayesian modelling. The Bayesian approach allows for the specification of prior distributions for the model parameters (here the between-group variance and the within-group error) for modelling the uncertainty. By combining this prior knowledge with the likelihood, inferences were made about the posterior distributions of the model parameters. Unlike frequentist methods that are generally poor at estimating between-group variances when there are a low number of groups [17], the Bayesian approach has been shown to produce reasonable estimates [18]. Three different prior distributions for both variance components (between-group and within-group) were used: (1) a flat distribution (prior density equal to 1 everywhere), (2) a non-informative Jeffreys prior (square root of the determinant of the expected information matrix), and (3) an inverse gamma distribution with parameters estimated from the data. Sampling from the posterior distribution was via an independence chain algorithm using SAS (the MIXED procedure), with 30,000 sampled values from the posterior distributions; 95% credible intervals are given as the 2.5% and 97.5% quantiles.

## 3. Results

### 3.1. Sample

Seven of the nine eligible study sites provided data. The numbers of eligible patients diagnosed with diabetes at each service ranged from 72 to 400 using PenCAT and ranged from 82 to 405 patients per service using Communicare. The numbers of eligible patients aged 35 years or more per service who were not diagnosed with diabetes ranged from 124 to 1397 using PenCAT and from 134 to 1079 using Communicare.

### 3.2. Size, Gender, and Age of Type 2 Diabetic Population

As shown in Table 1, the ICC estimates indicate a strong correlation between the PenCAT and Communicare data regarding the total number of patients with diabetes within services (ICC = 0.99), the number by gender (ICC = 0.98–0.99), and as a proportion by gender (ICC = 0.96). The correlation between PenCAT and Communicare data was very strong (ICC = 1.00) for patients aged 35 years and above, and moderate for the number and proportion of Type 2 diabetes patients per service in the 18–34 years age group (ICC = 0.65 and 0.80–0.82 respectively).

### 3.3. Testing for Type 2 Diabetes in Undiagnosed Patients

Table 2 indicates a strong to very strong correlation between service-level PenCAT and Communicare data regarding the number and proportion of people aged 18 years and over; or 35 years and over; who were not diagnosed with Type 2 diabetes; and who were tested for diabetes during a 12-month period (ICC = 0.94–1.00).

### 3.4. HbA1c Monitoring of Patients with Type 2 Diabetes

Table 2 shows the number and proportion of patients aged 18 years or over with Type 2 diabetes who had their HbA1c or plasma glucose monitored in a six-month period identified by PenCAT and Communicare. The ICCs for the numerator and denominator were both 0.99 and the ICC for the overall proportion was 1.00, indicating a strong correlation between the two systems in terms of reporting HbA1c monitoring rates.

### 3.5. Cholesterol Monitoring of Patients with Type 2 Diabetes

As shown in Table 2, there was a strong to very strong correlation (ICC = 0.99–1.00) between the number and proportion of patients aged 18 years or over with Type 2 diabetes who had their cholesterol monitored within a 12-month period according to PenCAT and Communicare.

## 4. Discussion

The whole-service data produced by PenCAT and Communicare for evaluating service-level testing and monitoring relating to Type 2 diabetes indicated a generally high degree of concordance between the data extracted by the two systems. Therefore, the less expensive or less complex option (depending on the individual circumstances of the service) is generally appropriate for monitoring diabetes testing and care, albeit with caveats as described below.

With regard to the number of patients requiring testing or monitoring, the Communicare software identified a slightly higher total number of patients with diabetes (1–15% more patients per service) than did PenCAT at six of the seven services. As the proportion of additional diabetes cases identified via Communicare was variable, this may be partly due to human error at the data entry stage.

The lowest level of concordance between the two datasets related to data categorised by age-group. The correlation was only moderate for the number and proportion of Type 2 diabetes patients per service in the 18–34 age group (ICC = 0.65 and 0.80–0.82 respectively). Given the lower concordance regarding age group, estimates of practice-wide compliance would be slightly less accurate in relation to the guideline, which refers to all of those aged 18 years and over. Concordance regarding time frames for testing (e.g., annually versus every three years) was not of concern. While Type 2 diabetes is more common in older age groups [11], it is becoming increasingly common in younger age groups in the Indigenous Australian population [19] and in general populations internationally [11]. Optimal management of diabetes in its earlier stages is important for minimising or delaying debilitating consequences such as limb amputation, blindness, and kidney failure [19]. Therefore, from a whole-community or population perspective, it is important that health services be able to accurately monitor their performance in caring for subgroups of patients. Further examination of the way in which each service records age-related data and the way in which each system calculates and extracts age is important in order to identify the source of the discrepancy.

### Limitations

The relatively small number of services and the eligibility criteria for the study limits the generalisability of the data to ACCHSs. The use of service-level data did not permit direct comparisons between the systems regarding concordance on an individual case level.

## 5. Conclusions

While different data management or patient management software systems should theoretically produce very similar data, this is not always the case. At a more detailed level, the extraction of data about subgroups of patients may not be interchangeable.

## Figures and Tables

**Table 1:** Intra-class correlation estimates of concordance between PenCAT and Communicare data for each service regarding the total number and proportion of patients with diabetes including by gender and age-group.

Type 2 Diabetes Patient Characteristic	Median ICC Estimate from Bayesian Approach (95% Credible Interval)		
	Prior = Flat	Prior = Jeffreys	Prior = Inverse Gamma
Total number of patients	0.99 (0.94, 1.00)	0.99 (0.95, 1.00)	0.99 (0.97, 1.00)
Number of females	0.99 (0.89, 1.00)	0.98 (0.92, 1.00)	0.98 (0.95, 1.00)
Number of males	0.99 (0.96, 1.00)	0.99 (0.97, 1.00)	0.99 (0.98, 1.00)
Number aged 18 to 34 years	0.70 (0.10, 0.96)	0.66 (0.12, 0.93)	0.66 (0.21, 0.89)
Number aged 35 years or more	1.00 (0.97, 1.00)	1.00 (0.98, 1.00)	1.00 (0.99, 1.00)
Proportion aged 18 to 34 years	0.82 (0.23, 0.98)	0.80 (0.30, 0.96)	0.80 (0.45, 0.94)
Proportion males	0.96 (0.73, 1.00)	0.96 (0.79, 0.99)	0.96 (0.86, 0.99)

**Table 2:** Intra-Class correlation coefficients for each study outcome.

	Median ICC Estimate from Bayesian Approach (95% Credible Interval)		
	Prior = Flat	Prior = Jeffreys	Prior = Inverse Gamma
Proportion of patients aged >35 years without a diagnosis of diabetes who were tested between 1 July 2012 and 30 June 2013 via a random plasma glucose test.			
Numerator	1.00 (0.98, 1.00)	1.00 (0.98, 1.00)	1.00 (0.99, 1.00)
Denominator	0.94 (0.64, 0.99)	0.94 (0.71, 0.99)	0.94 (0.81, 0.98)
Proportion	0.98 (0.84, 1.00)	0.98 (0.88, 1.00)	0.98 (0.92, 0.99)
Proportion of patients with Type 2 Diabetes aged >18 years who had their HbA1c recorded in a six-month period.			
Numerator	0.99 (0.95, 1.00)	0.99 (0.96, 1.00)	0.99 (0.98, 1.00)
Denominator	0.99 (0.94, 1.00)	0.99 (0.95, 1.00)	0.99 (0.97, 1.00)
Proportion	0.99 (0.91, 1.00)	0.99 (0.93, 1.00)	0.99 (0.96, 1.00)
Proportion of patients with Type 2 Diabetes aged >18 years who had their cholesterol recorded in a 12-month period.			
Numerator	1.00 (0.97, 1.00)	1.00 (0.98, 1.00)	1.00 (0.99, 1.00)
Denominator	0.99 (0.94, 1.00)	0.99 (0.95, 1.00)	0.99 (0.97, 1.00)
Proportion	1.00 (0.97, 1.00)	1.00 (0.98, 1.00)	1.00 (0.98, 1.00)
